# Modeling brain macrophage biology and neurodegenerative diseases using human iPSC-derived neuroimmune organoids

**DOI:** 10.3389/fncel.2023.1198715

**Published:** 2023-06-05

**Authors:** Jonas Cerneckis, Yanhong Shi

**Affiliations:** ^1^Department of Neurodegenerative Diseases, Beckman Research Institute of City of Hope, Duarte, CA, United States; ^2^Irell and Manella Graduate School of Biological Sciences, Beckman Research Institute of City of Hope, Duarte, CA, United States

**Keywords:** microglia, iPSCs, brain organoids, macrophages, Alzheimer's disease, stem cells, brain diseases

## Introduction

Tissue-resident macrophages (TRMs) are specialized myeloid cells that adapt to the local microenvironment and perform both core macrophage functions, such as phagocytosis and immune surveillance, as well as tissue-specific roles (Troutman et al., 2021). The identity of TRMs is established by a combination of their ontogeny (or lineage) and the surrounding tissue environment that provides distinct signaling cues to educate TRMs toward more specialized functions, such as synaptic pruning by microglia (Butovsky and Weiner, 2018; Prinz et al., 2019; Troutman et al., 2021; Paolicelli et al., 2022). Mechanistically, signal-induced transcription factor activity leads to tissue-specific chromatin remodeling and enhancer activation superimposed on core macrophage gene expression programs (Lavin et al., 2014; Troutman et al., 2021). However, human cell-based experimental systems to probe individual subtypes of TRMs, such as those of brain macrophages, as well as delineate the molecular mechanisms underlying TRM specialization are largely lacking. In this Opinion, we propose a platform of induced pluripotent stem cell (iPSC)-derived neuroimmune organoids to establish the diversity of human cell-based brain TRM models and study their roles in tissue homeostasis and disease.

## Brain macrophage identity and diversity

The brain macrophage population is composed of parenchymal microglia and border-associated macrophages (BAMs), also known as central nervous system (CNS)-associated macrophages (CAMs), that together maintain homeostasis of the CNS and its surrounding tissues (Gosselin et al., 2017; Butovsky and Weiner, 2018; Kierdorf et al., 2019; Prinz et al., 2019; Troutman et al., 2021). Microglia develop from the yolk sac erythromyeloid progenitors during primitive hematopoiesis and populate the brain parenchyma early in development (Butovsky and Weiner, 2018; Ginhoux and Garel, 2018; Paolicelli et al., 2022). Although the microglial population is maintained by cell proliferation with no input from peripheral myeloid cells under homeostatic conditions, microglia display substantial spatial and temporal heterogeneity (Grabert et al., 2016; Butovsky and Weiner, 2018; Friedman et al., 2018; Hammond et al., 2019; Young et al., 2021; Paolicelli et al., 2022). For example, cerebellar microglia exhibit increased debris clearance as compared to forebrain microglia as well as depend on colony-stimulating factor 1 (CSF-1) signaling, whereas forebrain microglia survive CSF-1 depletion (Ayata et al., 2018; Kana et al., 2019). Moreover, lipid-droplet-accumulating microglia arise in aging, whereas disease-associated microglia (DAM) are characteristic of Alzheimer's disease (AD) (Deczkowska et al., 2018; McQuade and Blurton-Jones, 2019; Marschallinger et al., 2020; Silvin et al., 2022). BAMs comprise macrophages at the boundaries of the CNS, including meninges, vasculature, and the choroid plexus (Kierdorf et al., 2019; Mildenberger et al., 2022). Like microglia, BAMs originate from yolk sac erythromyeloid progenitors although the dynamics of BAM subtype specification and maintenance vary (Goldmann et al., 2016; Utz et al., 2020; Masuda et al., 2022). Whereas meningeal and choroid plexus macrophages are established prenatally, perivascular macrophages attain their full identity postnatally, when the Virchow-Robin space of their residence is established (Masuda et al., 2022). Moreover, while meningeal and perivascular macrophages are stably maintained into adulthood, choroid plexus macrophages are gradually replaced by peripheral bone marrow-derived cells (Goldmann et al., 2016; Prinz et al., 2019; Van Hove et al., 2019). BAMs also exhibit transcriptional heterogeneity, presumably indicating functional specialization to support their tissues of residence (Mrdjen et al., 2018; Li et al., 2019; Van Hove et al., 2019). The importance of tissue-specific signaling for maintaining distinct TRM populations in the brain is exemplified by microglial but not BAM dependence on tumor growth factor β (TGF-β) signaling (Thion and Garel, 2020; Utz et al., 2020; Brioschi et al., 2023). Importantly, TGF-β signaling promotes expression of the spalt-like transcription factor 1 (*SALL1*), a master regulator of microglia identity. *SALL1* is not expressed in other macrophage populations except for Kolmer's epiplexus cells that populate the choroid plexus and might, in fact, be a subtype of microglia (Butovsky et al., 2014; Buttgereit et al., 2016; Van Hove et al., 2019; Troutman et al., 2021; Brioschi et al., 2023). Interestingly, culturing microglia *ex vivo* leads to downregulation of *SALL1* expression and a substantial loss of microglia gene expression signatures (Gosselin et al., 2017).

## Species-specific divergence of murine and human microglia

It is well-established that murine and human microglia exhibit considerable species-specific divergence, hindering therapeutic development targeting microglia (Smith and Dragunow, 2014; Galatro et al., 2017; Geirsdottir et al., 2019). For example, human microglia exhibit higher transcriptional heterogeneity than do murine microglia as well as distinct immune function- and aging-associated gene expression signatures (Galatro et al., 2017; Geirsdottir et al., 2019). Moreover, risk factor genes implicated in brain diseases, such as AD, that are associated with microglial functions are poorly conserved between rodents and humans (Mancuso et al., 2019; Wightman et al., 2021). A key microglial surface receptor triggering receptor expressed on myeloid cells 2 (*TREM2*) shares < 60% of amino acid identity between murine and human variants (Mancuso et al., 2019). Likewise, gene expression programs of mouse DAM and human AD microglia (HAM) implicated in AD progression share few similarities with each other (Srinivasan et al., 2020). Given these species-specific differences between murine and human myeloid cell biology, there is a great need to develop robust human cell-based models that could faithfully recapitulate human brain macrophage biology.

## Human iPSC-derived macrophages and microglia

Since its inception, the iPSC technology has offered an almost unlimited access to *in vitro* models of human brain cells that are otherwise difficult to obtain from primary human brain tissue (Takahashi et al., 2007; Yu et al., 2007; Shi et al., 2017; Li et al., 2018; Li and Shi, 2020; Tong et al., 2021). Various protocols to differentiate macrophage- and microglia-like cells (iMGs) from iPSCs have been developed (Abud et al., 2017; Lee et al., 2018; McQuade et al., 2018; Pocock and Piers, 2018; Hasselmann and Blurton-Jones, 2020; Tang et al., 2022; Washer et al., 2022). Notably, iPSC differentiation into iMGs entails mesodermal specification and transition of the differentiating cells through an erythromyeloid-like progenitor, reminiscent of primitive hematopoiesis (Buchrieser et al., 2017; Lee et al., 2018). Embryonic-like origin of iMGs indicates their applicability to study microglial and BAM biology, especially given that peripheral macrophages arising via definitive hematopoiesis do not fully attain microglial identity when transplanted into the mouse brain and exhibit distinct gene expression profiles, such as high levels of apolipoprotein E (*APOE*) expression (Bennett et al., 2018). However, embryonic iMG ontogeny is insufficient to establish their brain macrophage identity. Indeed, iMGs cultured in isolation exhibit limited expression of key microglial markers *SALL1* and the transmembrane protein 119 (*TMEM119*), indicating that such iMGs have not yet attained bona fide microglial identity (Hasselmann et al., 2019). To overcome this limitation, iMGs have been successfully transplanted into the rodent brain to derive chimeric mouse models (Hasselmann et al., 2019; Mancuso et al., 2019; Svoboda et al., 2019; Fattorelli et al., 2021). Transplanted iMGs exhibit complex morphology and increased expression of key microglial genes, including *SALL1* and *TMEM119*, indicating the presence of correct intrinsic programs in iMGs that can be activated by the appropriate environment (Hasselmann et al., 2019). Although chimeric mouse models enable studying the interactions between human microglia and mouse brain cells, the implications of modeling human-to-mouse cell-to-cell interactions remain to be clarified. It is thus highly desired to develop human cell-based three-dimensional (3D) models of the brain tissue, so that iMGs could both mature to attain their full identity and establish interactions with other brain cell types that are of human origin.

## Achieving iPSC-derived brain macrophage diversity using regionally-patterned neuroimmune brain organoids

**Figure 1:**
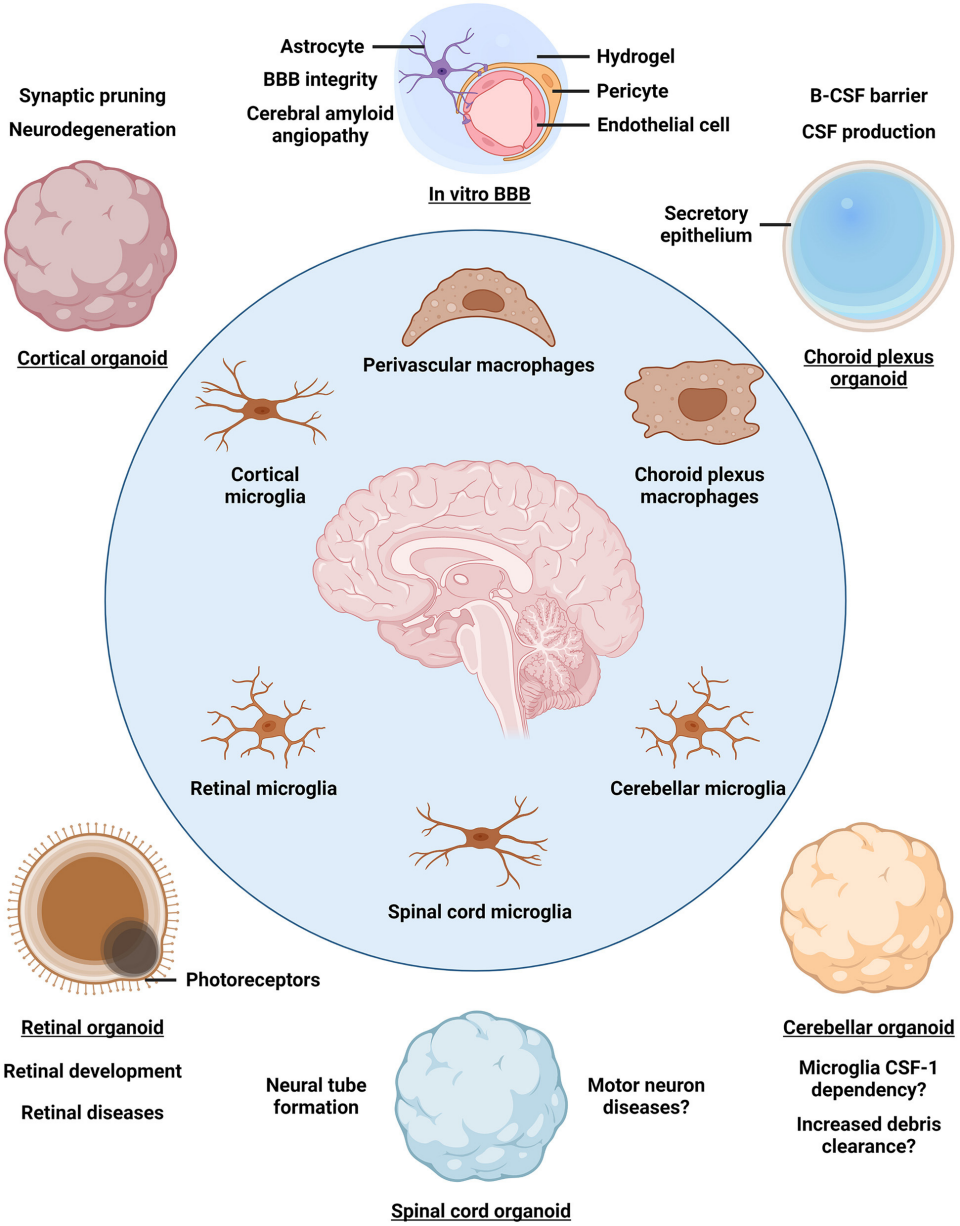
Organoid models for studying brain macrophage diversity. The identity of parenchymal microglia and border-associated macrophages is established by a combination of ontogeny and niche-specific environment that further educates brain macrophages to acquire tissue-relevant functionality. Incorporating induced pluripotent stem cell-derived macrophages into brain organoids patterned toward different brain regions may reveal niche-specific contributions to macrophage identity and function as well as enable disease modeling. BBB, blood-brain barrier; B-CSF barrier, blood-cerebrospinal fluid barrier; CSF, cerebrospinal fluid.

Brain organoids (BOs) are iPSC-derived 3D self-organizing assemblies of brain cell types and are used to model human brain development and disease (Lancaster and Knoblich, 2014). Given that myeloid cells arise from the mesoderm, whereas BOs are derived from the neuroectodermal lineage, various strategies to obtain iMG-containing neuroimmune organoids have been developed (Abud et al., 2017; Ao et al., 2021; Popova et al., 2021; Xu et al., 2021; Cakir et al., 2022). A simple and well-controlled approach to introduce iMGs into BOs involves iMG differentiation from iPSCs followed by iMG seeding onto BOs and infiltration into the neural tissue, although other protocols have been developed as well (Abud et al., 2017; Xu et al., 2021; Cakir et al., 2022; Zhang et al., 2023). So far, neuroimmune organoids have primarily been established using BOs derived by unguided differentiation, which yields organoids resembling a mixture of multiple brain regions, as well as using cortical BOs (Abud et al., 2017; Ao et al., 2021; Xu et al., 2021; Cakir et al., 2022; Jin et al., 2022). Carefully crafted guided differentiation approaches can be used to obtain BOs that resemble distinct brain regions and associated tissues, such as the forebrain, cerebellum, retina, choroid plexus, and others (Lullo and Kriegstein, 2017; Pellegrini et al., 2020; Sridhar et al., 2020; Nayler et al., 2021; Lee et al., 2022). Introduction of iMGs into such regionally-patterned BOs would provide a distinct environment to educate iMGs, so that diverse brain macrophage populations may be established (Figure 1). For example, cortical, cerebellar, retinal, and spinal cord neuroimmune organoids may be used to educate iMGs toward different microglia subtypes, whereas choroid plexus neuroimmune organoids may promote the identity of choroid plexus macrophages. Moreover, 3D blood-brain barrier (BBB) spheroids and *in vitro* BBB models may be used to educate iMGs toward perivascular macrophage identity (Cho et al., 2017; Blanchard et al., 2020). Having established regionally-patterned neuroimmune organoids with their distinct iMG populations, in-depth analysis of iMG morphology, functionality, response to stimulation, and gene expression programs would reveal how tissue residency promotes iMG specialization and how it compares to that of *in vivo* brain macrophage specialization. Do iMGs exhibit increased phagocytic activity in cerebellar neuroimmune organoids as compared to iMGs in cortical neuroimmune organoids? Do iMGs resemble distinct choroid plexus macrophage states, including Kolmer's epiplexus cells, in choroid plexus neuroimmune organoids? Do iMGs regulate cerebrospinal fluid production in choroid plexus neuroimmune organoids? These and other questions could be addressed by using the diverse neuroimmune organoid platform to elucidate the homeostatic roles of brain macrophages.

## Modeling inflammation and brain diseases using neuroimmune organoids

In addition to studying the homeostatic roles of brain macrophages, neuroimmune organoids can be used to reveal macrophage phenotypes in the context of brain diseases (Figure 1). As the sole immune cells of the brain parenchyma, microglia mount an inflammatory response upon stimulation, which may be protective under normal conditions, but becomes excessive and detrimental in a diseased brain, causing widespread inflammation (Lyman et al., 2014). Indeed, neuroinflammation is considered a hallmark of AD and other brain diseases (Lyman et al., 2014; Kinney et al., 2018; Leng and Edison, 2021). Exposing neuroimmune organoids to bacterial-derived lipopolysaccharide, amyloid β, physical injury, or virus infection leads to iMG activation, indicating a common response to an inflammatory stimulus (Abud et al., 2017; Ao et al., 2021; Xu et al., 2021; Cakir et al., 2022). Regionally-patterned neuroimmune organoids may be derived from iPSCs of patients carrying disease mutations or genetic risk factors, such as *APOE4*, and tailored to define iMG roles in distinct processes associated with disease progression. For example, how do iMGs respond to vascular amyloid seeding in perivascular neuroimmune organoids, mimicking cerebral amyloid angiopathy characteristic of AD? Importantly, neuroimmune organoids may help clarify how activated microglia shift from playing protective roles at early stages of neurodegenerative diseases to exacerbating tissue damage by sustained pro-inflammatory cytokine secretion and gliosis as the disease progresses (Deczkowska et al., 2018; Kinney et al., 2018; Leng and Edison, 2021). Another common feature of neurodegenerative diseases is the breakdown of the BBB, leading to peripheral monocyte-derived macrophage infiltration into the brain (Fani Maleki and Rivest, 2019; Silvin et al., 2022). To model peripheral macrophage infiltration, neuroimmune organoids may be co-cultured with primary monocyte-derived macrophages isolated from the donors' blood. In this way, the interactions between brain-resident microglia and peripheral macrophages as well as their different roles in disease progression may be clarified. Indeed, a recent study indicates that the transcriptional cluster of DAM in neurodegeneration is, in fact, comprised of protective parenchymal microglia and detrimental inflammatory macrophages infiltrating into the brain (Silvin et al., 2022). Therefore, elucidating the contributions of different macrophage populations to disease progression may inform therapeutic development targeting neuroinflammation.

## Discussion

Signaling from the tissue microenvironment is indispensable for establishing the TRM identity. Therefore, developing an array of iPSC-derived neuroimmune organoids resembling different brain regions and structures will provide a platform for studying brain macrophage diversity using iMGs. Moreover, neuroimmune organoids will enable modeling of human-specific cell-to-cell interactions between iMGs and other brain-resident cell types, so that novel molecular pathways that establish brain macrophage identity, specialization, and dysfunction in disease may be uncovered. We anticipate that the iPSC-derived iMG and organoid platforms will be widely applied to better define microglia and BAM states, perform high-throughput drug screening, and clarify disease-associated phenotypes. It will be important to compare any findings obtained from neuroimmune organoids to the transcriptomes of primary human brain macrophages to determine whether iMGs in neuroimmune organoids faithfully recapitulate human macrophage biology (Bian et al., 2020). Harnessing the human iPSC-based neuroimmune organoid technology will be especially beneficial for studying polygenic and sporadic diseases, such as AD, that cannot be easily recapitulated in animal models. In addition to the neuroimmune organoids discussed in this Opinion, further advances to the BO technology may enable modeling of meningeal structures and their resident dural and leptomeningeal macrophages (Kierdorf et al., 2019; Mildenberger et al., 2022), defining the roles of peripheral immune cells (Pasciuto et al., 2020; Zhang et al., 2022; Chen et al., 2023) and non-cellular factors (Chen et al., 2021; Liu et al., 2022) in microglia development and disease, and clarifying the transcriptomic and functional sexual dimorphism of microglia (Hanamsagar et al., 2017; Thion et al., 2018; Kelava et al., 2022). Finally, the same principles of using specialized neuroimmune organoids to establish a distinct iMG environment could also be applied to brain tumor organoids to better define the diversity and function of microglia and macrophages in the context of brain cancer (Hambardzumyan et al., 2016; Keane et al., 2021).

## Author contributions

JC wrote the manuscript and prepared the figure. JC and YS edited and revised the manuscript. All authors contributed to the article and approved the submitted version.
